# Overview on Adjunct Ingredients Used in Hydroxyapatite-Based Oral Care Products

**DOI:** 10.3390/biomimetics7040250

**Published:** 2022-12-19

**Authors:** Joachim Enax, Bennett T. Amaechi, Erik Schulze zur Wiesche, Frederic Meyer

**Affiliations:** 1 Research Department, Dr. Kurt Wolff GmbH & Co. KG, Johanneswerkstr. 34 36, 33611 Bielefeld, Germany; 2 Department of Comprehensive Dentistry, School of Dentistry, University of Texas Health San Antonio, 7703 Floyd Curl Drive, San Antonio, TX 78229-3900, USA

**Keywords:** toothpaste, hydroxyapatite, lactoferrin, xylitol, zinc, allantoin, bisabolol, hyaluronic acid

## Abstract

Hydroxyapatite, Ca_5_(PO_4_)_3_(OH), is a biomimetic active ingredient, which is used in commercial oral care products such as toothpastes and mouthwashes worldwide. Clinical studies (in vivo) as well as in situ and in vitro studies have shown the preventive effects of hydroxyapatite in various field of oral care. In some products, hydroxyapatite is combined with other active ingredients, to achieve an additional antibacterial effect or to promote gum health. This review analyzes the efficacy of six selected natural and nature-inspired ingredients that are commonly used together with hydroxyapatite. These additional actives are either antibacterial (lactoferrin, xylitol, and zinc) or promote gum health (allantoin, bisabolol, and hyaluronic acid). A systematic literature search was performed, and all studies found on each ingredient were analyzed. In summary, all analyzed ingredients mentioned in this review are well described in scientific studies on their beneficial effect for oral health and can be used to expand the preventive effect of hydroxyapatite in oral care products.

## 1. Introduction

Oral care products such as toothpastes and mouthwashes are used to prevent oral diseases such as caries and gingivitis [1,2]. They usually contain various ingredients, such as remineralizing agents, antibacterial agents, abrasives, thickeners, humectants, preservatives, surfactants, and flavors [1]. Other ingredients can be added for sensitivity relief or tooth whitening [1,3,4,5].

In recent years, the use of biomimetic ingredients in oral care has become more relevant [6,7,8,9]. An example of a globally used biomimetic ingredient in oral care products is particulate hydroxyapatite, Ca_5_(PO_4_)_3_(OH), a synthetic calcium phosphate mineral that is inspired by the natural enamel crystallites [10,11]. Hydroxyapatite shows good biocompatibility when used in oral care products, and it is also used as a food additive [12,13]. Various studies have shown its efficacy in different areas, such as caries prevention and the reduction of dentin hypersensitivity. The preventive effects of hydroxyapatite used in oral care products have already been published extensively [7,14,15,16,17,18].

In oral care products, hydroxyapatite can be used as a sole active ingredient [19] (in toothpastes besides other “excipients” such as abrasives, surfactants, etc.) or can be combined with other natural or nature-inspired ingredients, e.g., antibacterial ingredients and ingredients for gum care, with the goal to provide safe and efficient oral care formulations for all age groups.

The aim of this review is to present and discuss studies analyzing the efficacy of six selected ingredients functioning either as antibacterial (lactoferrin, xylitol, and zinc) or caring/moisturizing agents (allantoin, bisabolol, and hyaluronic acid) that are used as adjunct ingredients in commercial hydroxyapatite-based oral care products.

## 2. Literature Search

Six adjunct ingredients that are used in hydroxyapatite-based oral care products (e.g., in Karex and Bioniq^®^, Dr. Kurt Wolff GmbH and Co. KG, Bielefeld, Germany) were selected based on their antibacterial or caring/moisturizing properties for this review. Note that some of the analyzed ingredients are also used as hydroxyapatite adjuncts in oral care products of other brands/companies.

The literature search was carried out using PubMed. The following search terms were used to identify the relevant studies:

“*X* AND (toothpaste OR dentifrice OR mouthwash OR mouth rinse)”, with *X* = lactoferrin, xylitol, zinc, allantoin, bisabolol, and hyaluronic acid OR hyaluronate (note that hyaluronate, e.g., sodium hyaluronate, is the salt of hyaluronic acid). All results until 19 October 2022 were included in this review. For lactoferrin, allantoin, bisabolol, and hyaluronic acid/hyaluronate, all abstracts were screened. Because of the high number of results for xylitol and zinc (Table 1), only the most recently published review articles of these two ingredients were screened; additionally, we refer to literature reviews/textbooks. For the search and screening of studies in oral care for lactoferrin, allantoin, bisabolol, and hyaluronic acid/hyaluronate, the following exclusion criteria were applied: not in the scope of this review (e.g., studies performed not in the field of oral care/dentistry or studies where the focus was not on the respective active ingredient searched), review articles, articles in other languages than English, case reports, and animal studies. The most relevant studies analyzing the efficacy of the six mentioned ingredients in oral care products/formulations were selected and analyzed by the authors.

## 3. Overview on Adjunct Ingredients Used in Hydroxyapatite-Based Oral Care Products

**Figure 1:**
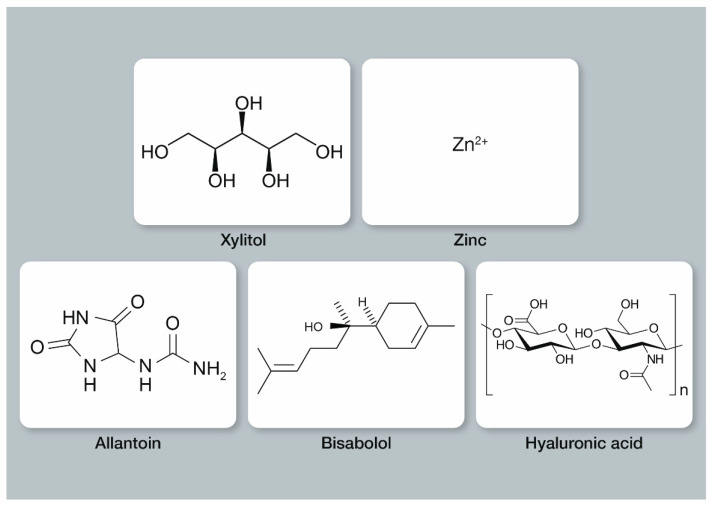
Structural formulas of the analyzed ingredients in this review used in combination with hydroxyapatite in oral care products (structural formulas according to ref. [20]; due to its complexity, the structure of lactoferrin is not shown; details have been published e.g., by Baker et al. [21]).

The literature search showed that zinc was the ingredient with the highest number of results (2026), followed by xylitol (244) and hyaluronic acid/hyaluronate (52) (Table 1). Details on each ingredient will be presented below. The structural formulas of the ingredients are depicted in Figure 1.

### 3.1. Antibacterial Ingredients

#### 3.1.1. Lactoferrin

Lactoferrin is an 80 kDa bilobal glycoprotein (connected by an α-helix), which can bind iron [21,22,23,24]. However, also different conformations of this protein exist that have ribonuclease-activity (cleaving RNA) but do not bind iron [25]. Binding iron leads to a confirmation-change from apolactoferrin to hololactoferrin [26]. Due to its iron-binding and RNase properties, lactoferrin shows antibacterial, antifungal, and antiviral properties [22,23,27]. Natural saliva contains approximately 8 µg mL^−1^ lactoferrin, and it can also be found in breast milk and tear fluid [28]. Lactoferrin for commercial use is usually isolated from bovine milk [22]. Human and bovine lactoferrin have been shown to have a high sequence-similarity with comparable antibacterial, antifungal, and antiviral properties [29].

*Studies in oral care:*

Lactoferrin is often used in oral care products in combination with other agents (e.g., other salivary enzymes such as lysozyme, or active oxygen), for an antibacterial effect, plaque reduction, and the prevention of gingivitis [23,30,31,32,33,34]. Lactoferrin-containing oral care products have been already successfully tested in children to reduce the levels of *Streptococcus mutans* in saliva [35]. Additionally, an in vitro study showed that lactoferrin reduces the attachment of early colonizers of the teeth, such as *Streptococcus gordonii* [36]. This helps to reduce bacterial biofilm-growth, thus preventing dental caries and gum diseases. Nagano-Takebe et al. showed that a lactoferrin layer can inhibit the initial bacterial colonization on titanium [37]. The authors concluded that covering implant-surfaces with lactoferrin can help to reduce periimplantitis. However, an in vitro study showed that a lactoferrin-containing toothpaste did not show superior antibacterial effects compared to a toothpaste containing the anionic surfactant sodium lauryl sulfate (SLS) [38]. In addition, Pizzo et al. could not find an inhibition of plaque regrowth by using a mouthwash with lactoferrin, lactoperoxidase, and lysozyme [39]. In patients with xerostomia, products with lactoferrin have been tested [40,41,42]. It is also part of some artificial saliva formulations [43]. An in situ/in vitro study by Jager et al. showed erosion-protective properties by using toothpaste with proteins (e.g., lactoferrin) [44]. For more information on lactoferrin in oral care, see reviews [27,28].

#### 3.1.2. Xylitol

Xylitol, C_5_H_12_O_5_, is a sugar alcohol that most oral bacteria cannot metabolize (Figure 1) [45]. It can be isolated from birch and beech trees as well as from corncobs, and it is safe for children [46]. Different modes of actions of xylitol have been described, e.g., bacteriostatic action and a reduction of the acid production from sucrose in bacteria biofilms [45]. While most sugars (carbohydrates, C*_x_*(H_2_O)*_y_*) can be metabolized by oral bacteria (glycolysis/fermentation) for energy-production (in form of adenosine triphosphate/ATP), this is not the case for xylitol. In brief, xylitol is actively taken up by oral bacteria, e.g., *Streptococcus mutans*, and this activates cellular mechanisms that should result in energy-production (ATP); however, most oral bacteria are not able to convert C-5-sugars to ATP at a level that is needed for cellular growth. Moreover, these cellular processes require energy, and the accumulation of C-5 sugars in bacteria cells inhibits glycolytic enzymes, leading to an energy-deficiency. Consequently, bacterial growth and acid production are inhibited, which is beneficial for caries prevention [47].

Xylitol is used not only in toothpastes and mouthwashes but also in products such as chewing gums and lozenges. Xylitol acts as a non-cariogenic sweetener in oral care products, where it stimulates salivary flow and increases the calcium and bicarbonate contents of saliva, thereby contributing indirectly to tooth remineralization and caries prevention. A recent meta-analysis shows the efficacy of xylitol in caries prevention, and xylitol was found to be more efficient than sorbitol and mannitol. It was concluded that the frequent use of xylitol is beneficial for oral health [48]. For more information on xylitol in oral care, see Limeback et al. and Fejerskov et al. [45,46].

#### 3.1.3. Zinc

Zinc is an important trace element in the human body with many different functions (Figure 1) [49]. Zinc salts have been used as antibacterial ingredients in toothpastes and mouthwashes for a long time, and different zinc salts are used for this purpose, e.g., zinc citrate, zinc chloride, and zinc PCA (the zinc salt of l-pyrrolidone carboxylate [50]) [1,51].

Zinc salts are mainly used to reduce dental plaque, inhibit dental plaque formation, and prevent gingivitis and halitosis [52]. Additionally, zinc has calculus-preventing/-reducing properties, mainly because of its antibacterial properties [1,45]. Calculus reductions up to 50% have been shown using zinc-containing toothpastes [1]. Notably, it has been described that zinc salts (in contrast to other antibacterial agents, such as stannous salts and chlorhexidine) do not stain the tooth surface [53]. Zinc ions can also be incorporated into the apatite lattice (Zn^2+^ as substituent for Ca^2+^ [54]), forming a zinc hydroxyapatite that can be used as an active ingredient in oral care products [55]. Interestingly, zinc is occurs naturally in saliva and dental plaque [56].

Zinc can be taken up into bacterial cells, where it inhibits the glycolytic enzymes, thus inhibiting bacteria metabolism, which leads to reduced bacterial growth. Zinc is a component of saliva and of diet, and it exhibits high substantivity in the oral cavity [17]. For more information on zinc salts in oral care, see Brading et al. and Marsh et al. [53,57].

### 3.2. Ingredients for Gum Care

#### 3.2.1. Allantoin

Allantoin, C_4_H_6_N_4_O_3_, is a heterocyclic organic molecule often used in skincare products, including products for babies (Figure 1) [58]. It can be synthesized, but there are also some natural sources (specific plants and food) [58]. Allantoin is used for different purposes, e.g., in oral care and skincare formulations [58]. Suzuki et al., have shown that allantoin can suppress the production of inflammatory cytokines by immune cells in human gingival fibroblasts *in vitro*. This helps to control inflammation in gingival cells [59].

*Studies in oral care:*

Magaz et al. showed that toothpastes and mouthwashes with allantoin, chlorhexidine, and dexpanthenol improved the periodontal disease index of patients with gingivitis [60]. A gel based on allantoin, chlorhexidine, and dexpanthenol improved wound healing [61]. Additionally, an allantoin-containing gel reduced postoperative pain and inflammation after tooth extraction [62]. Lopez-Lopez et al. demonstrated that an allantoin-containing gel is more effective in the reduction of pain and inflammation than a bicarbonate-based mouthwash after tooth extraction [63]. Allantoin was also found to influence cellular responses positively [64].

#### 3.2.2. Bisabolol

α-(−)-bisabolol (hereafter denoted as bisabolol [65]) is an organic molecule (a sesquiterpene alcohol) and a natural component of chamomile oil (*Matricaria* spp.) (Figure 1) [65,66,67]. It has been shown that bisabolol is the predominant constituent of *Matricaria chamomilla* [68]. It can also be found in other plants, e.g., *Eremanthus erythropappus* and *Smyrniopsis aucheri* [65]. Bisabolol has anti-inflammatory, anti-irritant, and antibacterial effects and is used in numerous dermatological and cosmetic products [65,68].

*Studies in oral care:*

Amora-Silva et al. showed that a bisabolol-containing mouthwash improved wound healing and reduced pain after oral surgery, and it has been proposed to be an alternative to chlorhexidine to be used after oral surgeries [66]. Additionally, the reduction/prevention of oral mucositis in patients undergoing chemotherapy (radiation therapy and systemic chemotherapy) by a commercially available mouthwash based on chamomile has been reported [69]. Notably, no burning or unwanted effects were documented after the application of the mouthwash, which is an important characteristic for an oral care product for this patient group [69]. Additionally, in an in vitro study it was shown that a combination of bisabolol and tea tree oil has an antibacterial effect against *Solobacterium moorei* (clinical isolates), which is associated with halitosis. Forrer et al. tested different concentrations of bisabolol and its antibacterial effect on different halitosis-associated oral bacterial strains. They were able to show that bisabolol (in combination with tea oil) was able to reduce their growth-rate even at low concentrations [70].

#### 3.2.3. Hyaluronic Acid

Hyaluronic acid, (C_14_H_21_NO_11_)*_n_*, is a glycosaminoglycan with various applications, e.g., in skin care (Figure 1) [71]. It is found not only on the skin but also in human saliva [72]. It acts as moisturizer by binding human cells and lubricating tissues [73].

*Studies in oral care:*

Hyaluronic acid and its salts (e.g., sodium hyaluronate) are used in oral care products, often in combinations with other ingredients (e.g., chlorhexidine), for plaque reduction and plaque control [74,75,76,77,78]. Several studies have shown that hyaluronic-acid-containing formulations can be used in the treatment and prevention of gingivitis/periodontitis [79,80]. For example, in combination with hydrogen peroxide, a hyaluronic-acid-containing mouthwash showed gingivitis-reducing effects [81]. Abdulkareem et al. showed that hyaluronic-acid-containing mouthwash was as effective as chlorhexidine in the reduction of gum bleeding; however, chlorhexidine showed a higher antibacterial effect than hyaluronic acid [82]. A mouthwash with hyaluronic acid and cetylpyridinium chloride was shown to be as effective as a chlorhexidine mouthwash in reducing plaque accumulation [83]. Furthermore, hyaluronic-acid-containing formulations reduced complications after tooth extractions [84,85]. However, Zorrilla et al. showed that the use of a chlorhexidine-chitosan gel resulted in better healing than with the use of a hyaluronic acid gel after oral surgery [86]. A mouthwash with hyaluronic acid, vitamin E, and triamcinolone acetonide can be used to treat oral mucositis [87]. This is in line with other studies on oral mucositis [88,89,90]. Other areas of application of hyaluronic-acid-containing formulations include the treatment of oral lichen planus [91,92,93], recurrent aphthous stomatitis [94], use in patients with implants [95], use in salivary substitutes [96], the reduction of symptoms after biopsy procedures [97,98], and use in denture-induced ulcerations [99]. Furthermore, Dong et al. described the use of antibacterial hyaluronic acid/chlorhexidine hydrogels [100].

### 3.3. Additional Ingredients

Hydroxyapatite can also be combined with other ingredients, e.g., with natural or nature-inspired substances [101], although the efficacy for some of them may be less documented in the literature. For example, a thorough in vitro screening of various alternative compounds regarding their antibacterial effect was published by Cieplik et al. [102].

## 4. Discussion

The biomimetic active ingredient hydroxyapatite is used in various fields of oral care [7,14,15,16,18,103]. It remineralizes early caries lesions [104,105,106,107], reduces the initial bacterial attachment to enamel similar to 0.2% chlorhexidine [19], and acts as buffer and a calcium and phosphate reservoir in biofilms [108]. To extend these preventive effects, hydroxyapatite can be used together with lactoferrin, xylitol, zinc, allantoin, bisabolol, and/or hyaluronic acid to achieve an antibacterial effect and to prevent/reduce gingivitis. In general, these combinations can be used in different oral care products (toothpaste, mouthwash, oral gel, etc.). However, each product has to be developed individually to avoid unintended interactions of the actives with other components of the formulation. Moreover, the stability, solubility, sensory properties, etc., of the actives in each product are important as well. The results of our search show that zinc salts have been described in more studies than the other analyzed active ingredients. A limiting factor in using, e.g., lactoferrin and hyaluronic acid in oral care products might be the relatively high price compared to “standard” ingredients of oral care products. Lactoferrin, zinc, allantoin, bisabolol, and hyaluronic acid are mainly used in oral care products for the prevention/treatment of periodontal diseases but also for other purposes (see above). Xylitol is mainly used to support caries prevention [46]. Xylitol is also used in chewing gums. It is important to mention that potential adjunct ingredients to hydroxyapatite are not limited to the ones discussed in this review because there are many other natural or natural-inspired active ingredients that can be used for various purposes [101,102]. A main goal in using such hydroxyapatite and adjunct ingredient(s) combinations in oral care is to provide safe and efficient alternatives to ingredients with potential side effects, such as tooth staining (chlorhexidine [109], stannous chloride/fluoride [110]), the risk of dental fluorosis in children (fluoride) [111], the potential risk of bacterial resistances (chlorhexidine) [112], or cytotoxic effects (cocamidopropyl betaine, sodium lauryl sulfate, and fluoride) [113]. Interestingly, the preventive spectrum of hydroxyapatite can be extended by the described adjunct ingredients (e.g., wound healing etc.; see above). Thus, various patient groups may benefit, for example, patients with periodontal diseases and patients with xerostomia.

This review has some limitations, which will be discussed hereinafter. It focuses on the search of studies analyzing the selected adjunct ingredients, and most tested formulations do not contain hydroxyapatite. There are, however, some studies analyzing formulations containing hydroxyapatite combinations (e.g., hydroxyapatite with lactoferrin [114], hydroxyapatite with xylitol [115,116,117], and hydroxyapatite with zinc (with zinc included in the apatite lattice [55] and zinc salts as an adjunct additive [8,116])). For example, Nocerino et al. studied the effect of hydroxyapatite particles functionalized with lactoferrin. They found the combination of hydroxyapatite and lactoferrin to be effective against different bacterial strains (gram-positive and gram-negative) but also to be more anti-inflammatory compared to lactoferrin alone [114]. Consequently, future studies should analyze potential synergistic effects of combined hydroxyapatite and known or novel adjunct ingredients. Another limitation is that many found that studies tested not only the efficiency of the sole ingredient but the effect of a whole formulation, e.g., hyaluronic acid with chlorhexidine [100], or lactoferrin with other salivary enzymes [40]. Note that hydroxyapatite itself has also been tested without any other ingredients, e.g., by Cieplik et al., and hydroxyapatite was shown to be a calcium and phosphate source and an acid buffer in bacterial biofilms in vitro [108]. Fabritius-Vilpoux et al. have shown the formation of mineral–mineral bridges between hydroxyapatite particles and enamel surfaces in vitro [11,118], and Kensche et al. studied the reduction of the initial bacterial colonization by hydroxyapatite in situ [19].

## 5. Conclusions

In conclusion, this review shows that there is good evidence that the reviewed adjunct ingredients, i.e., lactoferrin, xylitol, zinc, allantoin, bisabolol, and hyaluronic acid, can increase the oral disease preventive scope of hydroxyapatite-based oral care products.

## Figures and Tables

**Table 1:** Total results of the PubMed search of selected ingredients that are used as adjunct ingredients in hydroxyapatite oral care products (for details on the search see Section 2).

Ingredient	Total Number of Results	Number of Included Studies	Excluded Studies
lactoferrin	34	16	Not in the scope of this review: 13 Review articles: 3 Articles in other languages than English: 1 Case reports: 1 Animal studies: 0
xylitol	244	Due to the high number of studies, evaluation will be referred to other reviews	
zinc	2026	Due to the high number of studies, evaluation will be referred to other reviews	
allantoin	13	5	Not in the scope of this review: 3 Review articles: 0 Articles in other languages than English: 4 Case reports: 0 Animal studies: 1
bisabolol	10	3	Not in the scope of this review: 6 Review articles: 0 Articles in other languages than English: 1 Case reports: 0 Animal studies: 0
hyaluronic acid/hyaluronate	52	27	Not in the scope of this review: 15 Review articles: 6 Articles in other languages than English: 3 Case reports: 0 Animal studies: 1

## Data Availability

Not applicable.
